# Saxagliptin: A potential doping agent? A randomized, double‐blinded, placebo‐controlled, and crossover pilot study in young active men

**DOI:** 10.14814/phy2.15515

**Published:** 2022-12-02

**Authors:** Nicolas Bourdillon, Philippe J. Eugster, Céline Vocat, Toan Nguyen, Gregoire Wuerzner, Eric Grouzmann, Grégoire P. Millet

**Affiliations:** ^1^ Institute of Sport Sciences University of Lausanne Lausanne Switzerland; ^2^ Service of Clinical Pharmacology Lausanne University Hospital, University of Lausanne Lausanne Switzerland; ^3^ Service of Nephrology and Hypertension Lausanne University Hospital, University of Lausanne Lausanne Switzerland

**Keywords:** catecholamines, cycling, DPP4, exercise, neuropeptide Y, performance

## Abstract

Neuropeptide Ys (NPYs) contribute to sympathetic‐adreno stimulation: NPY1‐36 potentiates the effects of catecholamines (CATs), whereas NPY3‐36 inhibits CAT release. We sought to investigate whether inhibiting dipeptidyl‐peptidase‐4 (DPP4), cleaving NPY1‐36 into NPY3‐36, leads to increased NPY1‐36 potentiating effects and reduced NPY3‐36 inhibitory effects on CATs, thereby improving endurance performance. Seven male participants (age 27 ± 3 years, BMI 23.1 ± 2.4 kg/m^2^) performed time‐to‐exhaustion cycling exercise at 95% of peak power output with either placebo, or saxagliptin, a DPP4 inhibitor. Oxygen consumption (V̇O_2_), heart rate variability, NPY1‐36, NPY3‐36, catecholamines, and lactate were measured at several time points before, during, and after exercise. With saxagliptin, DPP4 activity (12.7 ± 1.6 vs. 0.2 ± 0.3 U/L, *p* = 0.001; d = 10.7) was decreased at rest, while NPY3‐36 (1.94 ± 0.88 vs. 0.73 ± 0.22 pm; *p* < 0.001; d = 2.04) decreased and NPY1‐36 increased during exercise (2.64 ± 2.22 vs. 4.59 ± 2.98 pm; *p* < 0.01; d = 0.19). CATs were unchanged. Time‐to‐exhaustion was 32% higher with saxagliptin. The difference in time‐to‐exhaustion between placebo and saxagliptin was correlated with NPY1‐36 differences (R = 0.78, *p* < 0.05). Peak V̇O_2_ and other cardio‐respiratory values were not different, whereas peak NPY concentrations were higher with saxagliptin. DPP4 blockade improved performance, increased NPY1‐36, and decreased NPY3‐36 concentrations which may have potentiating effects on the influences of CATs. However, DPP4 is involved in many different actions, thus NPYs are one group of factors that may underly its performance‐enhancing effects; further studies are required to determine the exact mechanisms.

## 1 INTRODUCTION

Since the Ancient Olympics Games, athletes have sought to improve their performances using exogenic substances. Recent scientific knowledge indicates a neuropeptide as short as a few amino acids could be a candidate for the next performance‐enhancing drug. It is therefore important that the code to ban those drugs is continually updated, to list the latest substances discovered (World Anti‐Doping Code, 2021).

Neuropeptide Y (NPY) is a 36‐aminoacid peptide that contributes to the effects of sympatho‐adrenal stimulation (Allen et al., 1983). It is co‐secreted with norepinephrine (NE) and epinephrine (E) in the adrenal medulla and co‐stored with NE in peripheral sympathetic nerve endings (Ekblad et al., 1984). NPY operates as a neural and endocrine messenger and is associated with stress resilience (Wagner et al., 2016).

Physical exercise causes release of NPY and catecholamines (CATs); (Lind et al., 1994) so that their plasma concentrations rise simultaneously in an intensity‐dependent manner (Pedrazzini et al., 2003). During post‐exercise recovery, NE returns to basal values in less than 10 min whereas NPY concentrations remain high for at least 15 min (Lacroix et al., 1997).

NPY binds to Y1, Y2, Y4, and Y5 receptors; the effects of NE are potentiated by post‐synaptic Y1 receptor stimulation (Wahlestedt et al., 1985), whereas the effects of NE and acetylcholine are inhibited by pre‐synaptic Y2 receptor stimulation (Westfall et al., 1987). Once released from nerve endings, NPY1‐36 mainly binds to the Y1 receptor. Under the action of dipeptidyl‐peptidase‐4 (DPP4) (Wagner et al., 2016), NPY1‐36 is cleaved into NPY3–36 a Y2/Y5 agonist (Medeiros & Turner, 1996). In turn, NPY3‐36 is cleaved into NPY3–35, which is unable to bind to Y1, Y2, and Y5 receptors and may represent the major metabolic clearance product. NPY2‐36 is a Y2 agonist but its concentrations are minor in humans (Abid et al., 2009). In summary, NPY1‐36 potentiates the effects of CATs whereas NPY3‐36 inhibits CAT release and NPY3‐35 is ineffective.

CATs have well‐described ergogenic effects (Magkos & Kavouras, 2004) and performance‐enhancing effects and taking exogenic CATs‐agonists is a common doping strategy (Davis et al., 2008). Although many stimuli influence CAT release, the intensity of exercise is key. The highest plasma concentrations of CATs are measured during exercise at maximal intensity. Moreover, long‐term adaptations to high‐intensity training include increases in plasma concentrations of CATs and NPY (Rämson et al., 2012).

Given the ability of NPY1‐36 to potentiate the effects of CATs, we hypothesized that inhibiting the action of DPP4 would increase the potentiating effects of NPY1‐36 and reduce the inhibitory effects of NPY3‐36 on CATs; thereby potentially enhancing physical performance during high‐intensity exercise.

The present study follow‐up on our previous study (Eugster et al., 2022) whose initial aims were to assess the role of NPY1‐36 during exercise and the role of NPY3‐36 during recovery. The present study describes secondary outcomes on performance improvement in young active men who received a DPP4 inhibitor prior to maximal intensity exercise to exhaustion (Grouzmann et al., 2007).

## 2 METHODS

### 2.1 Participants

Inclusion criteria were that participants should be aged 18 to 30 years old, physically active, not overweight, non‐smokers, and medication free, no disease or injury at the time of the study nor during the preceding month.

Eight healthy males, who trained at least 3 h per week (running), volunteered for this study which was approved by the local ethics committee (CER‐VD, 2018–00569). They provided informed written consent before participation. Seven participants completed this study, one dropped out due to pill intake issues.

### 2.2 Experimental design

This study was randomized, double‐blinded, and placebo controlled. Participants underwent one familiarization and two experimental sessions with a standardized breakfast in the laboratory at 7am on each occasion. The familiarization session was performed 1 week prior to the first experimental trial.

During familiarization, a maximal incremental test to exhaustion was performed on a cycle ergometer to determine peak power output, maximal heart rate (HRmax), and maximal oxygen consumption (V̇O_2max_), as detailed elsewhere (Eugster et al., 2022). Briefly, the participants were equipped with a heart rate monitor (Polar H7, Polar electro, Kempele, Finland) to measure R‐R intervals from which the heart rate variability indices were computed. This heart rate monitor is reliable and has been validated previously (Hernández‐Vicente et al., 2021; Plews et al., 2017) and a facial mask (Hans Rudolph Inc.,) connected to a gas analyzer (Jaeger Oxycon Pro, Acertys Healthcare Ltd) for oxygen consumption (V̇O_2_), carbon dioxide production (V̇CO_2_), and minute ventilation (V̇E) measurements. The participants remained seated for 5 min on the ergometer at rest before warming up and pedaling at 60 W for 3 min. Then, increments of 1 W every 2 s were applied until the participant could no longer sustain a cycling frequency of 70 rpm despite the investigators' strong verbal encouragement, a plateau in V̇O_2_ was observed and the respiratory exchange ratio was above 1.1.

For the two experimental sessions performed in a random order, the participants received either 5 mg of saxagliptin (Onglyza, AstraZeneca) or placebo (mannitol) per os, administered the night before and on the morning of the two experimental sessions, separated by a two‐week washout period. Capsule preparation and randomization was performed by the hospital pharmacy before participants' recruitment. Capsule allocation codes remained in a sealed envelope opened after data collection ended. The experimental session under saxagliptin is denoted Gliptin whilst the placebo session is denoted Placebo. The dose of 5 mg of saxagliptin is recommended in diabetes treatment, this drug is commonly prescribed worldwide with negligible known side effects (Ali & Fonseca, 2013; Subrahmanyan et al., 2021). Special precautions were taken on the potential breach of antidoping regulations by asking the LAD ‐ Antidoping Laboratory of Lausanne, as well as on the potential health risks or side effects of the administered dose.

### 2.3 Procedures

All procedures are described elsewhere (Eugster et al., 2022). The two experimental sessions were performed similarly, except for drug ingestion. The participants wore the same equipment as during familiarization. They remained seated on the ergometer for 5 min before pedaling at 95% of their PPO until exhaustion despite the investigators' verbal encouragements. Time‐to‐exhaustion was the main cycling performance criterion (Hopkins et al., 2001).

Upon exercise cessation, the participants recovered in a supine position for 90 min. Data from the gas analyzer and the HR monitor were averaged over 30‐s successive windows during exercise and 1‐min successive windows during recovery. Root mean square of the successive R‐R interval differences (RMSSD) was computed on each window as an indicator of parasympathetic vagal modulation of HR (Force, 1996). Rate of perceived exertion (RPE) was evaluated at rest and upon exercise cessation (Borg, 1982).

The insertion of a peripheral venous catheter allowed the collection of blood samples for assay of DPP4, NPYs, and CATs (Bergmann et al., 2017; Eugster et al., 2022; Vocat et al., 2020) of 2.6 ml each. The first blood sample of each experimental session was collected in the supine position for determination of DPP4 activity expressed as unit (U) per L of plasma (1 U was 1 μmol of substrate cleaved per min). All other blood samples were collected in Li‐Hep Monovette tubes containing a mixture of proteases inhibitors to prevent ex‐vivo peptide degradation. Concentrations of NPYs and CATs were measured in each of these samples drawn at 1 and 5 min in the seated position on the ergometer, each minute during exercise and at 1, 2, 5, 10, 20, 30, 40, 50, 60, and 90 min during recovery.

All sessions were performed in an air‐conditioned room where average temperature was 25.3°C and humidity was 32.3%.

### 2.4 Statistics

Time‐to‐exhaustion in the Gliptin and Placebo conditions were compared using a pairwise Student's t‐test. Correlations between Gliptin‐to‐Placebo differences in time‐to‐exhaustion and in NPY concentrations changes were performed using Pearson's R coefficient. A two‐way ANOVA (time × condition) was performed to assess possible interactions between the effects of saxagliptin and the rest‐exercise‐recovery sequence. In all cases, the significance level was set at 0.05. *p*‐values are presented <0.05, <0.01, and <0.001. All results are presented mean ± SD except on the figures where data are plotted as mean ± SEM for better clarity. Cohen's d are given when possible for effect size assessment (negligible <0.2, medium >0.2 and <0.8, large >0.8). All analyses were performed using MATLAB (v.R2021a, The MathWorks Inc).

*A priori* computations of sample size were performed for two‐way ANOVA with interaction on NPY concentrations, using an effect size of 0.8 (large), an alpha of 0.05, and a power of 0.8 which resulted in a sample size of 6 and an actual power of 0.83 (G*Power v3.1.9.7).

## 3 RESULTS

Table 1 summarizes participants' data from the incremental exercise test (familiarization).

**TABLE 1:** Participants' characteristics.

Age (year)	Height (cm)	Weight (kg)	V̇Emax (L/min)	V̇O_2_max (ml/min/kg)	Peak power output (W)	HRmax (bpm)
27 ± 3	178 ± 6	73 ± 9	160 ± 26	50.2 ± 7.4	325 ± 36	180 ± 9

Abbreviations: HRmax, maximal heart rate; V̇Emax: maximal ventilation; V̇O_2_max, maximal oxygen consumption.

During the two experimental sessions, cycling exercise was performed at 95% peak power output corresponding to 309 ± 32 W. DPP4 activity was lower at rest in Gliptin than in Placebo (0.2 ± 0.3 vs. 12.7 ± 1.6 U/L; *p* < 0.001; d = 10.7). As expected, DPP4 blockade resulted in an NPY3‐36 decrease at rest in Gliptin compared to Placebo (−234%, *p* < 0.001, d = 2.04, paired *t*‐test).

**FIGURE 1:**
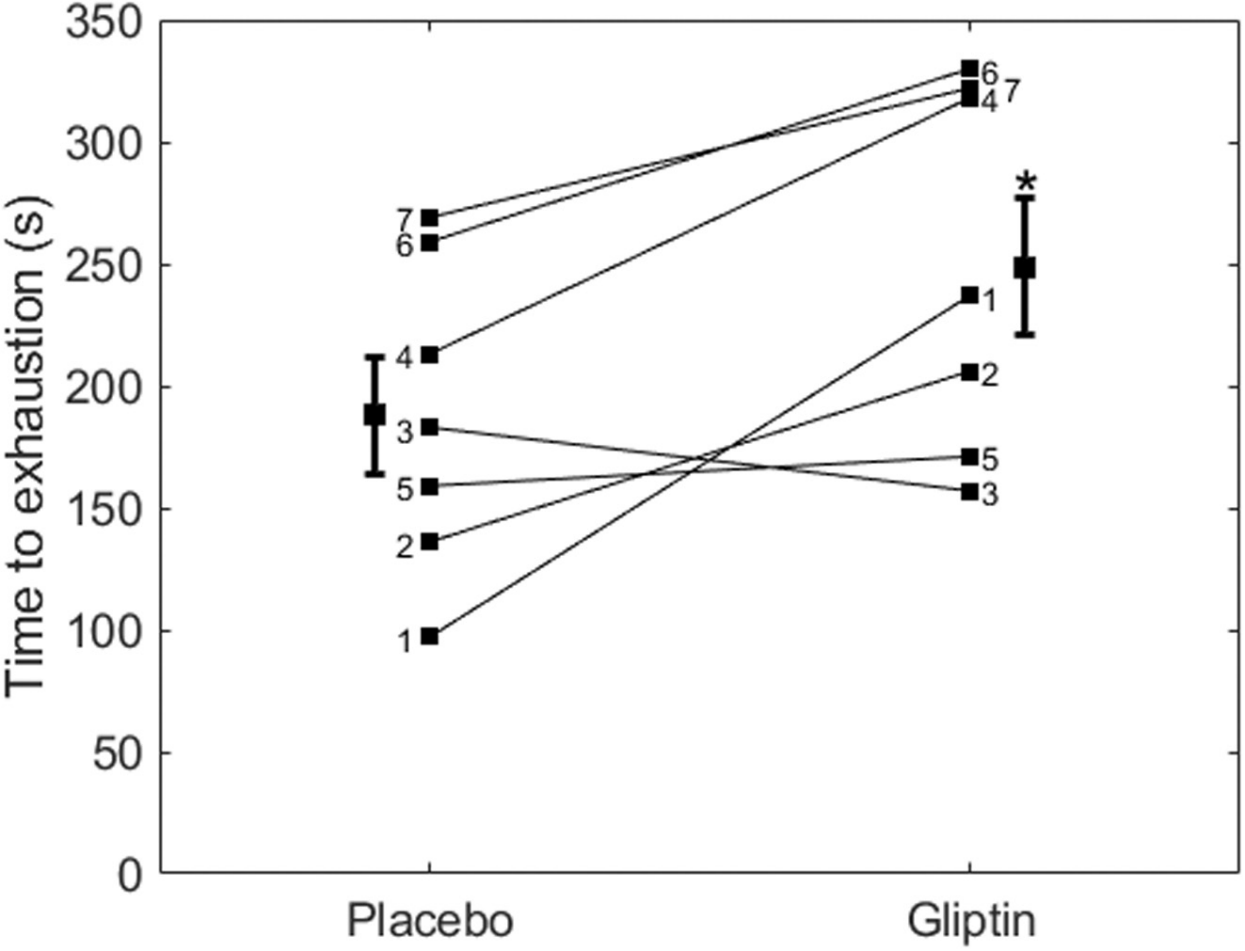
Time‐to‐exhaustion (in second) in placebo and gliptin conditions, and scatter plot of individual datapoints.

**FIGURE 2:**
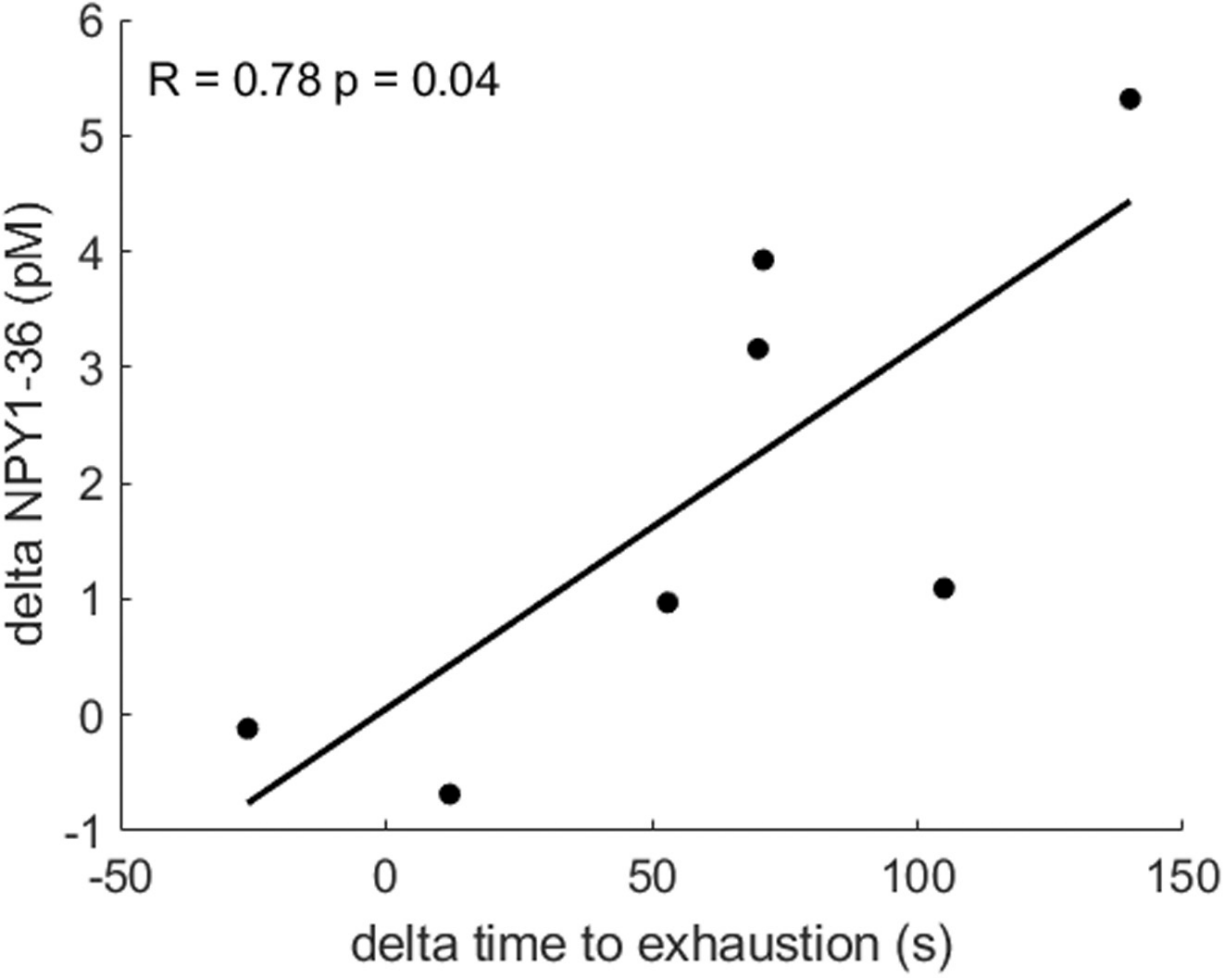
Pearson's correlation between differences in time‐to‐exhaustion and in NPY1‐36 plasma concentration between placebo and gliptin conditions.

Figure 1 shows a significant increase of 32% in time‐to‐exhaustion in the Gliptin condition compared to Placebo (d = 0.88). Figure 2 shows a significant correlation between the Placebo‐to‐Gliptin changes in time‐to‐exhaustion and in peak NPY1‐36 concentration.

**FIGURE 3:**
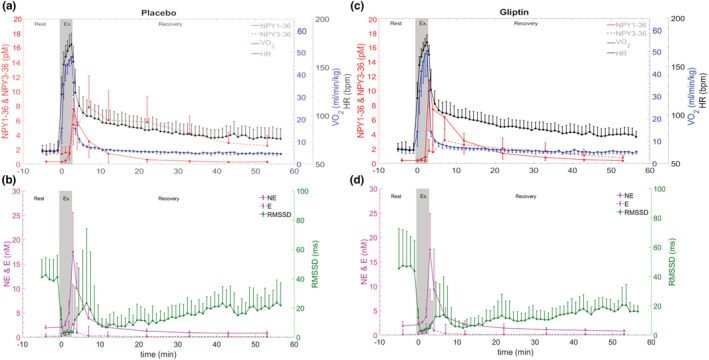
Time course of oxygen consumption (V̇O_2_), carbon dioxide production (V̇CO_2_), minute ventilation (V̇E), heart rate (HR), root mean square of the successive RR differences (RMSSD), NPY1‐36, NPY3‐36, norepinephrine (NE), epinephrine (E) during rest, exercise at 95% of peak power output and supine recovery. Panel a: NPY1‐36, NPY3‐36, V̇O_2_, and HR in the placebo condition. Panel b: NE, E, and RMSSD in the placebo condition. Panel c: NPY1‐36, NPY3‐36, V̇O_2_, and HR in the gliptin condition. Panel d: NE, E, and RMSSD in the gliptin condition.

Figure 3 illustrates the time course of V̇O_2_, HR, and plasma NPY1‐36, NPY3‐36, NE, and E concentrations at rest, during exercise, and recovery. Despite the increase in time‐to‐exhaustion (Figure 1), maximal values in V̇O_2_ and HR were reached during exercise in both Placebo and Gliptin. For better clarity, the significances are described below rather than in Figure 3.

DPP4 blockade had no significant effect on V̇O_2_, V̇E, or RMSSD, but slightly increased HR globally throughout the Gliptin condition (+4%; *p* < 0.05; d = 0.14 in Gliptin vs. Placebo). There was no exercise x condition interaction on any cardio‐respiratory variables (V̇O_2_, V̇E, HR, or RMSSD).

In both Placebo and Gliptin, V̇O_2_ and HR increased rapidly during the first minute of exercise and then kept increasing at a slower rate (*p* < 0.001 for both). No plateau was reached. During the first 3 min of recovery V̇O_2_ and HR decreased rapidly (*p* < 0.001 for both) before V̇O_2_ reached basal values, while HR remained higher than basal values (*p* < 0.05) and kept decreasing seemingly linearly.

Both DPP4 blockade (*p* < 0.01; d = 0.19) and exercise (*p* < 0.001; d = 1.70) increased NPY1‐36 concentrations, but there was no significant interaction (*p* = 0.07). The highest concentration was consistently found at the first minute of recovery, when NPY1‐36 was significantly higher in the Gliptin condition compared to Placebo (+52%; d = 0.65). At other time points, any differences between Gliptin and Placebo were not significant. NPY1‐36 remained significantly elevated for 10 min (fourth blood draw into recovery) after exercise cessation in both Gliptin and Placebo conditions.

Exercise led to an increased NPY3‐36 concentration compared to resting values (*p* < 0.001; d = 0.46) whilst DPP4 blockade decreased it (*p* < 0.001; d = 2.04), and there was an exercise x condition interaction (*p* < 0.001). During rest, exercise and recovery, NPY3‐36 concentrations were lower in the Gliptin condition compared to Placebo. During the first 20 min into recovery (first five blood draws of recovery), NPY3‐36 was higher than during rest and exercise in the Gliptin condition, but it was not as high as in the Placebo condition. NPY3‐36 concentrations returned to basal levels after 20 min in the Gliptin condition and 50 min in the Placebo condition.

As expected, both NE and E increased during exercise (*p* < 0.001; d = 1.62 and 1.27, respectively), but there was no effect of DPP4 blockade nor exercise x condition interaction. The highest concentrations in NE and E were measured 1 min into recovery. In both conditions, NE returned to basal levels at the fifth minute while E returned at the second minute into recovery.

As expected RMSSD decreased after exercise onset, but in a comparable way between Gliptin and Placebo. During recovery, RMSSD increased but did not return to basal values within 50 min of recovery, as shown in Figure 3.

As expected, lactate concentration increased post‐exercise, but no differences were observed between the Gliptin and Placebo conditions (Table 2, *p* = 0.07; d = 0.21, paired *t*‐test).

**TABLE 2:** V̇O_2_, HR, NPY1‐36, NPY3‐36, NE, and E at rest and during exercise in placebo and gliptin conditions.

	Rest		Exercise	
	Placebo	Gliptin	Placebo	Gliptin
V̇E (L/min)	12.8 ± 0.4	13.0 ± 1.1	127.7 ± 18.0	132.0 ± 29.4
% V̇Emax	‐	‐	80.3 ± 7.5	82.1 ± 9.3
V̇O_2_ (ml/min/kg)	6.2 ± 0.9	6.2 ± 1.2	50.6 ± 4.1	48.8 ± 10.2
% V̇O_2_max	‐	‐	98.6 ± 8.9	96.1 ± 9.0
HR (bpm)	63 ± 7	64 ± 6	176 ± 12	181 ± 6
% HRmax	‐	‐	96.0 ± 3.0	96.9 ± 1.9
La (mM)	1.3 0.6	1.4 ± 0.6	10.7 ± 2.4	11.9 ± 1.8
RPE	6.1 ± 0.3	6.1 ± 0.4	18.9 ± 1.6	18.2 ± 1.9
NPY1‐36 (pM)	0.35 ± 0.11	0.39 ± 0.11	2.64 ± 2.22	4.59 ± 2.98*
NPY3‐36 (pM)	1.35 ± 0.78	0.41 ± 0.20 *	1.94 ± 0.88	0.73 ± 0.22**
NE (nM)	2.00 ± 0.54	2.02 ± 0.64	8.43 ± 5.29	9.79 ± 5.23
E (nM)	0.23 ± 0.09	0.19 ± 0.05	1.15 ± 0.96	1.37 ± 0.73

*Note*: Resting values are averaged over the 5 min of rest. Exercise values are averaged over the last 30 s of exercise for V̇E, V̇O_2_, and HR; and are the last blood sample before cessation of exercise for La, NPY1‐36, NPY3‐36, NE, and E. Abbreviations: E, epinephrin; HR, heart rate; La, Lactate; NE, norepinephrine; NPY, neuropeptide Y; RPE, rate of perceived exertion; V̇E, minute ventilation; V̇O_2_, oxygen consumption. *p < 0.05, **p < 0.01 compared to Placebo.

## 4 DISCUSSION

The main result of the present study was that saxagliptin ingestion induced an improvement in cycling performance at maximal intensity. The positive correlation between the Placebo‐to‐Gliptin changes in time‐to‐exhaustion and in peak NPY1‐36 suggests that NPY1‐36, by potentiating NE, may cause this performance improvement. However, DPP4 is involved in numerous mechanisms beyond NPY, and the current study cannot rule out that factors we did not measure were involved. The specific assessments of NPY1‐36 and NPY3‐36 during exercise were performed using a unique newly validated technique (Eugster et al., 2022; Vocat et al., 2020). The present study is the first to report saxagliptin might be used as a potential doping agent, but caution is required because the 32% increase in time‐to‐exhaustion resulted in a smaller time‐trial performance improvement and may not translate into improved performance for elite athletes.

### 4.1 Effective blockade

The large reduction in DPP4 activity demonstrated that the administration of saxagliptin achieved effective blockade. Moreover, the two‐week wash‐out period was sufficient to restore basal DPP4 activity. This blockade resulted in a major decrease in NPY3‐36 concentrations, indicating that NPY1‐36 cleavage was severely blunted. In addition, at peak concentration, NPY3‐36 decreased by 68% whereas NPY1‐36 concentrations increased by 51%. To our knowledge, it is the first time that such changes have been observed during maximal intensity exercise when NPY1‐36 release is expected to be maximal.

### 4.2 Performance, NPY1‐36 and NPY3‐36

Performance improvement was correlated with the change in NPY1‐36 concentration under DPP4 blockade, which is consistent with our hypothesis that increasing NPY1‐36 improves maximal‐intensity performance by increasing its potentiating influences on the post‐synaptic effects of CATs. However, the present study cannot rule out the possibility that other mechanisms are involved in this improvement in performance. For example, there may also be an effect of decreased NPY3‐36 concentrations during DPP4 blockade, because a decrease in NPY3‐36 concentration may limit its inhibitory effect on release of NE, E, and NPY1‐36. However, since there was no significant increase in NE or E concentrations during DPP4 blockade, any NPY3‐36‐induced inhibitory effects are likely to have been secondary to the effects of increased NPY1‐36. Another possibility is that NE and E on the one hand and NPY1‐36 on the other, are stored in different vesicles and their respective release is differently affected by NPY3‐36 binding to pre‐synaptic Y2 receptors. This idea is supported by the different time courses of plasma CAT and NPY concentrations at the onset of submaximal exercise (Eugster et al., 2022).

During post‐exercise recovery there was a faster return to basal NPY3‐36 concentrations in the Gliptin condition (20 vs. 50 min in Gliptin vs. Placebo). Further, in accordance with previous studies, NE and E concentrations returned to baseline earlier than NPY1‐36 and NPY3‐36 concentrations (Eugster et al., 2022; Pernow et al., 1986), but there was no difference between Gliptin and Placebo conditions. Thus, it seems unlikely that Gliptin‐induced blockade of a pre‐synaptic inhibitory influence on CAT release was important during recovery.

Overall, performance would benefit from faster recovery from each bout of exercise in repeated sprints and intermittent sports (Dupont et al., 2010). However, since the present study investigated only one period of continuous aerobic exercise, further investigation on intermittent exercise will be required to test the potential ergogenic effect of saxagliptin under these conditions.

In addition to saxagliptin‐induced effects on NPYs, other potential effects of DPP4 inhibition must be considered. In particular, the potential effects of an increase in insulin blood concentrations are relevant because saxagliptin is prescribed for its glucoincretin effect in patients with type 2 diabetes. A better use of glucose with gliptin might in part explain the improved performance during strenuous exercise. It should be noted that saxagliptin was used only twice at the therapeutic dose before the onset of exercise in the present study. Nevertheless, future studies should examine the extent to which the effects of performance we have identified might reflect effects of saxagliptin on insulin.

### 4.3 Cardiovascular effects

Stimulation of β‐adrenergic receptors in the cardiovascular and respiratory systems has well‐described effects such as increased heart rate and myocardial contractility and blood flow redistribution toward skeletal muscle, as well as bronchodilation. The present study evidenced a slight chronotropic positive effect under DPP4 blockade with saxagliptin which may result from the potentiating effect of NPY1‐36 on the post‐synaptic effects of CATs. There was no significant difference in RMSSD between Placebo and Gliptin, as it would be expected because the effect on RMSSD can be attributed to the effect of maximal exercise intensity on vagal withdrawal (Fontolliet et al., 2018).

There was no measure of a potential cardiac inotropic effect or of changes in regional blood flow distribution in the present study, but DPP4 blockade may have influenced these responses. Potentiation of the post‐synaptic effects of NPY1‐36 on the action of CATs on β‐adrenergic receptors may have augmented cardiac contractility, while increased potentiating effects of NPY1‐36 on the actions of CATs on vascular β‐adrenergic receptors in skeletal muscle and α‐adrenergic receptors in non‐skeletal muscle vasculature may have improved distribution to exercising muscle and away from renal and splanchnic circulations by enhancing vasodilation and vasoconstriction, respectively. This should be assessed in future studies. On the other hand, V̇O_2_ and V̇CO_2_ were unchanged under DPP4 blockade indicating that if respiratory airflow were improved by enhanced bronchodilation, the improvement in performance was not due to increased V̇O_2_, which reached a plateau in maximal intensity exercise under Placebo and Gliptin conditions.

### 4.4 Skeletal muscle effects

β‐Adrenergic stimulation also improves exercise performance through enhanced skeletal muscle contractility, potentiation of peak force, and faster relaxation of some muscles (Cairns & Borrani, 2015). Potential inotropic and lusitropic effects of DPP4 blockade were not measured, but as performance improved and V̇O_2_ did not increase during exercise, it can be speculated that increased NPY1‐36 concentrations may potentiate the inotropic effect of CATs on skeletal muscles (Cairns & Borrani, 2015).

It is also possible that any effects of the blockade of DPP4 we used in the present study on incretins, improved glucose uptake and potentially increased glycolysis during maximal exercise (Aroor et al., 2014). Oxidative phosphorylation activity and mitochondrial efficiency may also be improved although this latter point remains unexplored. These effects would resemble the sport performance‐enhancing effects of β‐adrenergic agonists such as salbutamol or clenbuterol (Spann & Winter, 1995).

### 4.5 Limitations

DPP4 inhibitors may have a pleiotropic action through multiple peptides (Mulvihill & Drucker, 2014) whose degradation blockade may not only increase NPY1‐36 but also stimulate glucoincretin effects on insulin. While insulin is a clearly identified doping agent (World Anti‐Doping Code, 2021), the present study cannot exclude the possibility that the performance‐enhancing effects we identified were due to effects of incretins or other mechanisms rather than those attributed directly to NPYs. Thus, future studies should assess each substrate in the context of improved performance following gliptin ingestion.

Seven participants is a relatively small sample size but was sufficient to demonstrate significant improvement in performance and a significant correlation between NPY1‐36 concentration and performance. However, this pilot study was conducted on male participants; future studies should include female participants and larger groups at different levels of training to test whether the presented findings can be extrapolated.

### 4.6 Clinical implication

The present study opens new perspectives about the role of NPY1‐36 and NPY3‐36 during physical exercise, yet many mechanisms remain to be elucidated. If confirmed on elite athletes, with such diverse effects, saxagliptin may be a potential doping agent for use in endurance sports. From a clinical point of view, the cardiac effect of increased NPY1‐36 concentrations may open the way to new cardiac therapy in patients with chronic heart failure (Kalla et al., 2020).

## 5 CONCLUSION

This study demonstrated that ingestion of the DPP4 antagonist saxagliptin, improved cycling performance at maximal intensity in healthy male individuals. This improved performance was associated with increased plasma NPY1‐36 and decreased NPY3‐36 concentrations and may be explained by potentiating effects of NPY on the actions of NE and E. However, future studies are needed to determine the specific mechanistic effects of saxagliptin on NPYs, incretins, and other substances that are important in skeletal muscle and myocardium during and after exercise and to confirm whether saxagliptin might be used as a doping agent.

## AUTHORS' CONTRIBUTION

Nicolas Bourdillon, Philippe J. Eugster, Céline Vocat, and Grégoire P. Millet designed the study. Nicolas Bourdillon, Philippe J. Eugster, Toan Nguyen, and Céline Vocat performed data collection and analysis. NB prepared the figures and wrote the manuscript. Philippe J. Eugster, Eric Grouzmann, Gregoire Wuerzner, and Grégoire P. Millet revised the manuscript. All authors approved the final version of the manuscript.

## FUNDING INFORMATION

No specific grant was received for this study.

## CONFLICTS OF INTEREST

The authors declare that they have no competing interests.

## CONSENT FOR PUBLICATION

Not Applicable.

## ETHICS STATEMENT

This study was approved by the Swiss ethics committee of Vaud, Switzerland (CER‐VD, 2018–00569). All participants provided informed written consent before participation.
